# Impacts of continuing education on Primary Health Care professionals—A scoping review protocol

**DOI:** 10.1371/journal.pone.0312963

**Published:** 2025-01-24

**Authors:** Laianny Krizia Maia Pereira, José Adailton da Silva, Ricardo A. de M. Valentim, Thaísa G. F. M. S. Lima, Nésio Fernandes de M. Junior, Alexandre R. Caitano, Tatyana Souza Rosendo

**Affiliations:** 1 Postgraduate Program in Family Health (RENASF), Federal University of Rio Grande do Norte, Natal, Rio Grande do Norte, Brazil; 2 Laboratory for Technological Innovation in Health (LAIS), Federal University of Rio Grande do Norte, Natal, Rio Grande do Norte, Brazil; 3 Department of Biomedical Engineering, Federal University of Rio Grande do Norte, Natal, Rio Grande do Norte, Brazil; Norbert Wiener University, PERU

## Abstract

**Introduction:**

Continuing Health Education is a strategy that integrates learning into the work process to transform health practices. Primary health care has proved to be a powerful space for consolidating continuing education, as it promotes reflection and learning based on the local singularities of the territory. Continuing health education is an important strategy for transforming the reality of Primary health care, reinventing work, and consequently changing practices.

**Objective:**

This study aimed to identify and analyze the evidence regarding the impact of continuing education for health professionals in Primary Health Care in Brazil.

**Method:**

This scoping review protocol that will be developed based on the Joanna Briggs Institute manual and guided by the Preferred Reporting Items for Systematic Reviews and Meta-Analyses Extension for Scoping Reviews (PRISMA-ScR). The databases included in the study will be MEDLINE/Pubmed, Scielo, Lilacs, EMBASE, and Web of Science. Google Scholar will be used as gray literature. Studies published between 2007 and 2024 will be included, and no language restrictions will be applied. The Rayyan management software will be used as a tool to support the selection of studies. Two independent researchers will carry out a paired review to ensure the relevance of the studies. The studies will be analyzed descriptively, which will be guided by the categorization of results into three large groups: a) thematic areas covered; b) methodologies used; c) work process indicators and/or health results.

**Discussion:**

Relevant aspects will be pointed out and interrelated in narrative form with the existing literature on the subject. Through the preparation of this protocol, it is aimed to identify to identify studies with evidence on the impact of continuing education in Primary Health Care in Brazil. This review could support the formulation of new professional training policies for PHC, contributing to the improvement of professional practices and the organization of work based on the needs of each territory.

## 1 Introduction

Continuing Health Education (CHE) consists of a strategy aimed at presenting a model of human training in health that integrates learning into the work process, with the aim of changing health practices and then improving their quality. This concept emerged in Latin America in the 1980s, disseminated by the Pan American Health Organization’s Human Resources Development program [1] in an effort to overcome training exclusively focused on technical skills and enable greater worker involvement in the health production process.

In the context of the Brazil’s National Health System (SUS, for its acronym in Portuguese), human training in health was posed as a challenge. This led Brazil’s Ministry of Health (MoH) to institute in 2004, the National Policy for Continuing Health Education (PNEPS). The PNEPS aims to promote actions to change everyday health practices, where workers would bring with them knowledge and experiences that would motivate the process of reflection on care practices, tailoring them to the actual needs of the population and in line with the SUS principles and guidelines [2]. The conception of CHE in the National Policy for Continuing Health Education transcends the pedagogical meaning, responding to a process of restructuring services in the face of the new demands of the model [3].

There are various methodological strategies for facilitating health workers’ learning. When thinking about CHE, one must primarily consider active teaching methods and aim for meaningful learning, i.e., taking into account the previous knowledge of people involved in the process [4]. It is a fact that continuing education seeks to overcome the traditional models of qualification and continuing education, in which activities were designed and developed in isolation from the actual needs of the services [5]. In addition, it envisages valuing work and workers, the active participation of practitioners in the teaching-learning process, and workplace transformations [6].

Primary health care (PHC) is a powerful space for consolidating continuing education, promoting reflection and learning based on different scenarios, taking into account local singularities [7]. Oliveira and colleagues [8], which obtained a Brazilian panel of Continuing Health Education based on official data collected in the 2nd cycle of the Primary Care Quality Improvement Program (PMAQ), observed that, in Brazil, 90% of the Family Health Teams claimed to participate in some CHE action. Therefore, despite its potential, CHE initiatives in primary care have not been appreciated and implemented. CHE needs to be legitimized as an educational model and policy in Brazil in order for the quality of management and care to be improved [9].

The current context of CHE in Brazil requires efforts aimed at articulation and institutional partnerships between service and teaching, education and work. In this sense, for its implementation, many challenges still need to be overcome, among them the greater incentive for the use of new technologies for health education and the establishment of a commitment to the new demands of public health [10]. The Brazilian states with the greatest progress in the implementation of CHE actions were those that included pedagogical innovations aimed at meaningful learning for work, among them, workshops aimed at the formation of multidisciplinary teams, as well as the planning of distance education activities using Information Communication Technologies (ICTs) [11].

Given the potential of continuing health education in qualifying professionals and, consequently, to improving the quality of care in primary settings, there is a gap in the literature regarding its effects on care practice in PHC. Therefore, it is necessary to carry out a study that seeks to understand the impacts of CHE aimed at primary care practitioners. Specifically, a study that intends to discover the topics most often covered, the methodological strategies most often used, and the work process and health outcome indicators used to measure the impacts achieved.

Therefore, this study aimed to present a scoping review protocol to identify and analyze the evidence regarding the impacts of continuing education for health professionals in the context of primary health care in Brazil.

## 2 Materials and methods

This study adopted the scoping review approach. For this, the method proposed by Joanna Briggs Institute–JBI will be used [12], as well as the extension of the checklist Preferred Reporting Items for Systematic Reviews and Meta-Analyses extension for Scoping Reviews (PRISMA-ScR–S1 File) [13]. The protocol for this review was registered with the Open Science Framework (DOI 10.17605/OSF.IO/784ED). This study does not directly involve human participants and ethical approval is not required.

The review protocol consists of six consecutive stages: (1) Definition and alignment of objective and research question; (2) selection of search terms; (3) definition of inclusion/exclusion criteria; (4) data extraction; (5) analysis of results; (6) presentation of results [14].

### 2.1 Stage 1: Definition and alignment of objective and research question

Considering the objective of this study, the guiding question was formulated according to the PCC mnemonic–(Population, Concept, Context) [5]:

P–Population (Health Professionals)

C–Concept (Continuing Education)

C–Context (Primary Health Care)

Therefore, the main question of the review is: What are the impacts of continuing education for PHC professionals? The secondary questions are: (i) What are the thematic area(s) addressed in the continuing education actions of health workers in PHC? (ii) What methodological strategies have been used in continuing education in the context of PHC? (iii) What work process and health outcome indicators were used to measure the impact of the strategy adopted?

Table 1 details the key concepts that will underpin the research question.

**Table 1:** Key concepts for the research question.

Health Professionals	Individuals that provide health services, whether as individual physicians or employees of health institutions and programs, trained or untrained health professionals, whether or not subject to public regulation [15]
Continuing Education	Educational programs that aim to inform individuals about recent progress in their particular field of interest. They do not lead to any advanced conventional position [13]
Primary Health Care	Essential health care based on practical, scientifically based, and socially acceptable methods and technologies, made available to all individuals and families in the community through their full participation and at a cost that the community and the country can afford, at every stage of its development, in a spirit of self-responsibility and self-determination. (Declaration of Alma-Ata—Pan American Health Organization, 2003) [13]

Source: own authorship, 2023.

### 2.2 Stage 2 –Selection of search terms

Based on the PCC mnemonic, the search strategies were designed according to each database, following keywords and three controlled health vocabularies: Health Sciences Descriptors (DeCS), Medical Subject Headings (MeSH), and ENTREE, together with Boolean operators "AND" and "OR," to obtain a wide scope of results in the different databases. Natural language (uncontrolled vocabulary) was also used to increase the sensitivity of the strategy. English was used to structure the research strategy since it is the main language used in scientific circles. Table 2 shows the descriptors used according to the PCC Mnemonic.

**Table 2:** Descriptors used according to PCC Mnemonic.

Synonyms/keywords
health personnel; health care providers; health care provider; healthcare providers; healthcare provider; healthcare provider; healthcare workers; healthcare worker; health care professionals; health care professional; health care professional.
permanent education in health; permanent education; continuing education; continuous learning; lifelong learning; life-long learning; life-long learnings; lifelong learning in health.
primary healthcare; primary care.

Source: Own authorship, 2023

The databases to be included in the study are MEDLINE/Pubmed, Scielo, Lilacs, EMBASE, and Web of Science. The choice of databases is justified by theirhigh credibility, i. e., index journals with a good academic reputation. In addition to being they are references for scientific data in the field of study of the research in question. Google Scholar will be adopted as gray literature. In the case of Google Scholar documents, the first 100 documents will be considered for analysis. Articles identified in the references of the studies selected from the searches carried out and related to this review’s objective will also be included.

To help build and organize the search strategy, the Extraction, Conversion, Combination, Construction, and Use (ECUs) model will be adopted [16]. It allows highly sensitive search strategies to be developed following a set of complementary steps. Hence, the search strategy will be adapted according to each database. Table 3 shows the standard search strategy.

**Table 3:** Standard search strategy.

P	“Health Personnel” OR “Health Care Providers” OR “Health Care Provider” OR “Health Care Provider” OR “Healthcare Providers” OR “Healthcare Provider” OR “Healthcare Provider” OR “Healthcare Workers” OR “Healthcare Worker” OR “Health Care Professionals” OR “Health Care Professional” OR “Health Care Professional”
C	“permanent education in health” OR “permanent education" OR "continuing education" OR “continuous learning" OR "lifelong learning" OR "life-long learning" OR "life-long learning” OR "life-long learnings" OR "life long learning" OR "life-long learnings" OR “lifelong learning in health”
C	“primary health care” OR “primary healthcare” OR “primary care”
Standard Search Strategy	(“permanent education" OR “Continuous Learning” OR “Lifelong Learning” OR “Life-Long Learning” OR “Life-Long Learning” OR “Life-Long Learnings” OR “Life Long Learning” OR “Life-Long Learnings” OR “Continuing Education”) AND (“Health Personnel” OR “Health Care Providers” OR “Health Care Provider” OR “Health Care Provider” OR “Healthcare Providers” OR “Healthcare Provider” OR “Healthcare Provider” OR “Healthcare Workers” OR “Healthcare Worker” OR “Health Care Professionals” OR “Health Care Professional” OR “Health Care Professional”) AND (“Primary Health Care” OR “Primary Healthcare” OR “Primary Care”)

Source: own authorship, 2023.

The search strategy was pre-tested on MEDLINE/PubMed for scientific literature and Google Scholar for gray literature, in august 2023, to check for data collection limitations related to the search strategy.

### 2.3 Stage 3 –Definition of inclusion/Exclusion criteria

The review will include publications that address continuing education for health professionals in the context of PHC, carried out in Brazil, which answers the question of the study. Being:

- Primary studies
- Gray literature: books, manuals, protocols, brief communications, dissertations and theses.

The survey will comprise works available in full and in electronic form, without language limitations and published between 2007 and 2024. This time frame is justified by covering the period of validity of Ministerial Ordinance No. 1,996 of August 20, 2007, which establishes the guidelines for the implementation of the National Policy on Continuing Health Education (PNEPS) [17].

Duplicate publications, letters to the editor, expanded abstracts, editorials, and unavailable articles, even after contacting authors, will be excluded.

### 2.4 Stage 4 –Data extraction

To guide the stages of study selection, the path proposed in the Preferred Reporting Items for Systematic Review and Meta-Analyses (PRISMA-ScR) will be used for both white and gray literature: (1) identification; (2) screening; (3) eligibility; and (4) inclusion, which will be detailed in the review flowchart.

Based on the inclusion and exclusion criteria, the title and abstract of all included studies will be analyzed. The search will take place systematically, in pairs, and independently, as recommended by the JBI Institute.(12) In the event of a disagreement, a third reviewer can intervene in the decision. The Rayyan management software will be used as a tool to support the selection of studies.

The studies selected by title and abstract will be recovered in their entirety and exported to a database in the Microsoft Excel® software. Two independent researchers will perform a paired review to ensure the relevance of the studies. A flowchart will then be drawn up containing information on the screening and selection of studies included and excluded from this scoping review based on the results presented for evaluation and synthesis.

A pilot test will be carried out prior to the selection by the review team to ensure that the peer selection process is aligned. The pilot test will follow the following criteria: two reviewers will evaluate the same random sample of 25 articles by evaluating titles and abstracts in a data source and selecting them selecting them using eligibility criteria. The team will then meet to discuss and resolve the discrepancies and to make the necessary changes to criteria and definitions. Data extraction will only begin when 75% or more similarity is obtained.

The data related to the included studies will be extracted by two independent reviewers using a data extraction form prepared by the authors (see S1 Appendix). The form includes the Title of the publication, Authors, Year of publication, Country, Language, Study design, Study population, Study Objective, and any data related to the research questions—topics covered, methodology used, work process indicators, and health indicators used.

Any disagreement between reviewers will be resolved through discussion and consensus or, if necessary, by a third reviewer.

To answer the questions in this review, evidence will be extracted from both types of literature on the impact of continuing education for health professionals in primary health care.

### 2.5 Stage 5 –Analysis of results

The mapped studies will be analyzed descriptively, using the information described on the form used to extract the data (supplementary material) and other characteristics that are relevant to the findings. The results of the studies will be categorized into three groups of analysis: (a) topics covered; (b) methods used; and c) work process and/or health outcome indicators (health mortality or morbidity).

### 2.6 Stage 6 –Presentation of results

The final report will include flowcharts, tables, and/or figures. The results will be presented to experts in the area for discussion and the construction of better evidence, aiming to qualify the preliminary findings.

### 2.7 Timeline

Table 4 presents the timeline for the development of the stages of this review

**Table 4:** Timeline of study.

Activities	2024					
	APRIL	MAY	JUNE	JULY	AUGUST	SEPTEMBER
**Pilot test**						
**Selection of studies–analysis of titles and abstracts**						
**Selection of studies–analysis of studies in full**						
**Data extraction**						
**Data analysis and discussion**						
**Presentation of results–to experts**						
**Scope review submission**						

### 2.8 Dissemination of results

The aim is to submit the results for publication in a peer-reviewed journal and presentation at a scientific conference.

## 3 Discussion

Primary Health Care is configured as a powerful space for consolidating permanent education. And, given the potential of this strategy that integrates learning into the work process to transform health practices, and consequently, improve the quality of care, this study aims to present a scoping review protocol to identify and analyze the evidence regarding of the impacts of continuing education aimed at health professionals within the scope of Primary Health Care in Brazil.

This protocol aims to systematize methodological criteria to achieve the proposed objective. Relevant aspects will be pointed out and interrelated in narrative form with the existing literature on the subject. At the end of the scoping review, we hope to identify the impact of continuing health education on work processes and health indicators at the primary care level. The aim is also to identify gaps in knowledge that will encourage further studies on the subject. Drawing up this protocol will make it possible to carry out a study with high methodological rigor, quality, transparency, and precision, reducing the risk of bias.

This review may present limitations inherent to the search strategy when using only the term "health professionals", not including other terms related to the categories of health professionals, such as nurses and dentists, which may reduce its sensitivity in search for studies. However, this study could support the formulation of new professional training policies for PHC, contributing to the improvement of professional practices and the organization of work based on the needs of each territory.

## Supporting information

S1 File Completed PRISMA-ScR protocol. (PDF)

S1 Appendix Data extraction form. (DOCX)
