# Supplementary material for: Impacts of continuing education on Primary Health Care professionals—A scoping review protocol
Source: PLoS One. 2025 Jan 24;20(1):e0312963. doi: 10.1371/journal.pone.0312963 (PMC11761588; doi:10.1371/journal.pone.0312963)
Supplement: S1 File — (PDF) [file pone.0312963.s001.pdf]

PRISMA-P (Preferred Reporting Items for Systematic review and Meta-Analysis Protocols) 2015 checklist: recommended items to address in a systematic review protocol*

Section and topic	Item No	Checklist item	REPORTED ON PAGE #
**ADMINISTRATIVE INFORMATION**			
Title:			
Identification	1a	Identify the report as a protocol of a systematic review	The study is identified in the title as a protocol
Update	1b	If the protocol is for an update of a previous systematic review, identify as such	This is not an update to a previous revision
Registration	2	If registered, provide the name of the registry (such as PROSPERO) and registration number	Yes. The protocol was registered in the Open Science Framework (DOI 10.17605/OSF.IO/784ED).
Authors:			
Contact	3a	Provide name, institutional affiliation, e-mail address of all protocol authors; provide physical mailing address of corresponding author	This information is on page 1
Contributions	3b	Describe contributions of protocol authors and identify the guarantor of the review	Author contributions and funding are included in the manuscript submission system.
Amendments	4	If the protocol represents an amendment of a previously completed or published protocol, identify as such and list changes; otherwise, state plan for documenting important protocol amendments	The protocol does not represent an amendment to a previously completed or published protocol.
Support:			
Sources	5a	Indicate sources of financial or other support for the review	The financial disclosure is " Ministry of Health - Brazil. Project: 085/2021."
Sponsor	5b	Provide name for the review funder and/or sponsor	The financial disclosure is " Ministry of Health - Brazil. Project: 085/2021."
Role of sponsor or funder	5c	Describe roles of funder(s), sponsor(s), and/or institution(s), if any, in developing the protocol	The funder has no role in the development of this protocol
**INTRODUCTION**			
Rationale	6	Describe the rationale for the review in the context of what is already known	Yes. The review questions/objectives are on pages 5 and 6 of the
Objectives	7	Provide an explicit statement of the question(s) the review will address with reference to participants,	Yes. The key elements (e.g., population

		interventions, comparators, and outcomes (PICO)	or participants, concepts, and context) are on pages 5 and 6 of the manuscript.
**METHODS**			
Eligibility criteria	8	Specify the study characteristics (such as PICO, study design, setting, time frame) and report characteristics (such as years considered, language, publication status) to be used as criteria for eligibility for the review	Yes. The eligibility criteria is on page 10 of the manuscript.
Information sources	9	Describe all intended information sources (such as electronic databases, contact with study authors, trial registers or other grey literature sources) with planned dates of coverage	Yes, this information can be found on page 9 of the manuscript.
Search strategy	10	Present draft of search strategy to be used for at least one electronic database, including planned limits, such that it could be repeated	Yes. The full electronic search strategy is on Table 3-Standard search strateg
Study records:			
Data management	11a	Describe the mechanism(s) that will be used to manage records and data throughout the review	Yes. The mechanism(s) that will be used to manage records and data during the review are presented on page 11 and 12 of the manuscript.
Selection process	11b	State the process that will be used for selecting studies (such as two independent reviewers) through each phase of the review (that is, screening, eligibility and inclusion in meta-analysis)	Yes. The process for selecting sources of evidence is on page 11.
Data collection process	11c	Describe planned method of extracting data from reports (such as piloting forms, done independently, in duplicate), any processes for obtaining and confirming data from investigators	Yes. The methods of charting data are on page 12
Data items	12	List and define all variables for which data will be sought (such as PICO items, funding sources), any pre-planned data assumptions and simplifications	Yes. The list and definition are on Appendix 1.
Outcomes and prioritization	13	List and define all outcomes for which data will be sought, including prioritization of main and additional outcomes, with rationale	Yes. This information is contained on page 12 of the manuscript.
Risk of bias in individual studies	14	Describe anticipated methods for assessing risk of bias of individual studies, including whether this will be done at the outcome or study level, or both; state how this information will be used in data synthesis	Yes. This information is contained on page 13 of the manuscript.
Data synthesis	15a	Describe criteria under which study data will be quantitatively synthesised	Not applicable to this study
	15b	If data are appropriate for quantitative synthesis, describe planned summary measures, methods of handling data and methods of combining data from studies, including any planned exploration of consistency (such as I^2, Kendall's τ)	Not applicable to this study
	15c	Describe any proposed additional analyses (such as sensitivity or subgroup analyses, meta-regression)	Not applicable to this study
	15d	If quantitative synthesis is not appropriate, describe the type of summary planned	Yes. This information is contained on page 12 of the manuscript.
Meta-bias(es)	16	Specify any planned assessment of meta-bias(es) (such as publication bias across studies, selective reporting within studies)	Not applicable

Confidence in cumulative evidence	17	Describe how the strength of the body of evidence will be assessed (such as GRADE)	Yes. This information is contained on page 12 of the manuscript, Yes. This information is contained on page 12 of the manuscript, stage 6.

*** It is strongly recommended that this checklist be read in conjunction with the PRISMA-P Explanation and Elaboration (cite when available) for important clarification on the items. Amendments to a review protocol should be tracked and dated. The copyright for PRISMA-P (including checklist) is held by the PRISMA-P Group and is distributed under a Creative Commons Attribution Licence 4.0.**

From: Shamseer L, Moher D, Clarke M, Ghersi D, Liberati A, Petticrew M, Shekelle P, Stewart L, PRISMA-P Group. Preferred reporting items for systematic review and meta-analysis protocols (PRISMA-P) 2015: elaboration and explanation. BMJ. 2015 Jan 2;349(jan02 1):g7647.
