# Supplementary material for: Impacts of continuing education on Primary Health Care professionals—A scoping review protocol
Source: PLoS One. 2025 Jan 24;20(1):e0312963. doi: 10.1371/journal.pone.0312963 (PMC11761588; doi:10.1371/journal.pone.0312963)
Supplement: S1 Appendix — (DOCX) [file pone.0312963.s002.docx]

**APPENDIX S1**

**DATA EXTRACTION FORM**

| **STUDY CHARACTERISTICS** |
| --- |
| **Title of publication** |
| **Author(s)** |
| **Year of publication** |
| **Country** |
| **Language** |
| **Study design** |
| **Study population** |
| **Study objective(s)** |
| **Research question** |
| **INFORMATION RELATED TO THE RESEARCH QUESTIONS** |
| **Thematic area(s) covered in continuing education actions for health professionals in PHC.** |
| **Methodological strategies used in continuing education in the context of PHC.** |
| **Work process and health outcome indicators used to measure the impact of the strategy adopted.** |
